# Surviving institutional contradictions: routine data use practices at the subnational levels

**DOI:** 10.1093/oodh/oqag036

**Published:** 2026-09-18

**Authors:** Bigten Kikoba, Merina Luambano, Masoud Mahundi, Wilfred Senyoni

**Affiliations:** Department of Computer Science and Engineering, University of Dar es Salaam, Bagamoyo Road, Kijitonyama (Science), P.O. Box 33335, 14113, Dar es Salaam, Tanzania; Department of Information Systems and Technology, University of Dodoma, Benjamin Mkapa Road, P.O. Box 490, 41218, Dodoma, Tanzania; Department of Computer Science and Engineering, University of Dar es Salaam, Bagamoyo Road, Kijitonyama (Science), P.O. Box 33335, 14113, Dar es Salaam, Tanzania; Department of Informatics, University of Oslo, Ole-Johan Dahl's Building, Gaustadalléen 23B, P.O. Box 1080, Blindern 0316, Oslo, Norway; Health Information Systems Programme (HISP), number 22, Mazinde Street, Mikocheni B, code 14113, Dar es Salaam, Tanzania; Department of Computer Science and Engineering, University of Dar es Salaam, Bagamoyo Road, Kijitonyama (Science), P.O. Box 33335, 14113, Dar es Salaam, Tanzania; Health Information Systems Programme (HISP), number 22, Mazinde Street, Mikocheni B, code 14113, Dar es Salaam, Tanzania; Department of Computer Science and Engineering, University of Dar es Salaam, Bagamoyo Road, Kijitonyama (Science), P.O. Box 33335, 14113, Dar es Salaam, Tanzania; Health Information Systems Programme (HISP), number 22, Mazinde Street, Mikocheni B, code 14113, Dar es Salaam, Tanzania

**Keywords:** institutional work, data use, institutional contradictions, Routine Health Information System, DHIS2, Tanzania

## Abstract

Routine Health Information System data are widely promoted as a foundation for evidence-informed planning, supervision, and service improvement in low- and middle-income countries. Yet their use in decentralized health systems is often shaped by contradictory institutional logics. In this study, contradictory institutional logics refer to conflicting expectations about what actors should prioritize, how data should be used, and which resources are made available to do so. We examined how these contradictions shape routine data use at subnational levels of the Tanzanian health system and how local actors respond to them. We conducted a qualitative study in the Dodoma City Council and the Bahi District Council, Tanzania, between 2024 and 2025. Data were generated through 31 key informant interviews and nonparticipant observations across 12 purposively selected health facilities and two council health management teams. Participants included facility in-charges, health management information system focal persons, facility programme coordinators, and council program coordinators. Data were analyzed thematically using institutional theory as an interpretive lens. The findings from the two councils reveal that routine data use was not absent; rather, it was improvised within a contradictory institutional environment. Strengthening data use, therefore, requires more than technical deployment. It requires institutionalized procedures for review and supervision, broader analytic capacity at the facility level, better alignment between DHIS2 and GoT-HOMIS, and target-setting and feedback practices that are meaningful to local service realities. These findings are analytically relevant to similar decentralized settings, although they are not statistically generalizable beyond the study context.

## Introduction

Routine Health Information Systems (RHISs) are a central component of health system governance because they generate the recurring service data used for planning, supervision, and performance review (AbouZahr and Boerma 2005, Aqil et al. 2009). Routine data use, however, involves more than collecting or reporting numbers. In this manuscript, routine data use refers to the active analysis, interpretation, discussion, and application of routinely produced data for planning, monitoring, supportive supervision, and service improvement. Across many low- and middle-income countries, RHIS data remain underused despite major investments in digital platforms, dashboards, and reporting reforms (Amouzou et al. 2021, Hoxha et al. 2022). Tanzania reflects this broader challenge. Studies from Tanzania show persistent concerns around data quality, limited analytic capacity, uneven access to DHIS2, and weak organizational support for sustained data use at local levels (Rumisha et al. 2020, Mboera et al. 2021, Simba et al. 2022, Yusuph et al. 2024).

Existing research usually describes these problems as barriers, determinants, or capacity gaps (Karijo et al. 2021, Hoxha et al. 2022, Ayele et al. 2024, Kiwanuka et al. 2026). While useful, that language can flatten an important feature of the practice of local actors who are often responding simultaneously to competing expectations. Frontline health workers are expected to provide clinical care, generate high-quality data, enter data into digital systems, interpret indicators, present results in meetings, and act on those results, often without parallel increases in time, staffing, training, or decision space (Bossen et al. 2019, Bertelsen et al. 2024). We therefore frame the problem in terms of contradictory institutional logics (Berente and Yoo 2012). Institutional logics are the taken-for-granted assumptions, norms, and values that define legitimate action in organizations and fields (Friedland 2012). In this study, contradictory institutional logic refers to conflicts between at least three expectations: a service delivery logic that prioritizes immediate patient care; a reporting and accountability logic that emphasizes data completeness, timeliness, and compliance; and a digitalization and standardization logic that expects data to move through interconnected systems and standardized routines. By data use practices, we mean the coexistence of multiple locally specific ways of reviewing and acting on data, rather than one standardized subnational routine. By informal procedures, we mean uncodified local practices used in the absence of formal guidance, such as attaching data review to other meetings, selecting *ad hoc* indicators for community feedback, hand-modifying tally sheets, or manually reconciling reports across systems.

The study draws on institutional theory and the concept of institutional work to explain not only what constrains routine data use but also how local actors respond. Institutional work refers to the purposive actions through which actors create, maintain, or disrupt institutions (Lawrence and Suddaby 2006). This perspective is useful in RHIS research because it moves beyond asking whether data are used and instead examines how healthcare workers and managers improvise practices that make data usable under imperfect conditions. The study therefore asks: *What contradictory institutional logics hinder effective routine data use at the local level, and what strategies do local actors employ to navigate them?*

Our contribution is three-fold. First, we provide a clearer conceptualization of contradictory institutional logics in relation to routine data use. Second, we offer a richer empirical account of how these contradictions are experienced in two Tanzanian councils, drawing on interview and observational material and respondent excerpts from the key informants. Third, we show that local actors are not merely constrained by institutions; they also actively rework them through practical, situated responses. The claims of this article are limited to the Tanzanian cases studied, but they may offer analytically transferable insights for similar decentralized health systems.

## Theoretical framework

Institutional theory explains how rules, norms, and shared meanings shape organizational behavior and become taken for granted over time (Meyer et al. 1977). As explained by Meyer et al. (1977), institutionalization, in this sense, is the process through which a practice becomes expected, legitimate, and routinely reproduced. In RHIS terms, routine data use would be institutionalized when analyzing and acting on data are not a special project activity or donor-driven exercise but an ordinary, supported part of service management (Sahay et al. 2019).

We combined two strands of this literature. The first is the institutional logics perspective, which draws attention to competing value systems and expectations within organizations (Berente and Yoo 2012). The second is institutional work, which focuses on the purposive actions of actors who create, maintain, or disrupt these arrangements (Lawrence et al. 2011, Senyoni 2021). We use contradictory institutional logics to identify points where expected action is undermined by conflicting assumptions or arrangements. For example, staff may be held accountable for data-informed action while budgets remain controlled elsewhere, or digital systems may be deployed as if standardized data work already exists, even when training and workflows remain highly uneven.

To organize the empirical analysis, we used the Performance of Routine Information System Management (PRISM) framework as a sensitizing structure because it distinguishes organizational, behavioral, and technical determinants of RHIS performance (Aqil et al. 2009). PRISM was not used as a rigid coding frame into which all findings were forced. Rather, it provided the first layer of categorization, after which institutional theory was used to interpret why those determinants appeared as contradictions and how actors responded through institutional work. This combined approach allowed us to distinguish between isolated obstacles and broader conflicts between expectations, roles, and system arrangements.

## Material and methods

### Study design and setting

We conducted a qualitative case study in two councils in Dodoma Region, Tanzania: Dodoma City Council and Bahi District Council. The study formed part of the District of Excellence (DoE) project, a 4-year initiative (2022–25) aimed at strengthening subnational health information systems. Because the research was embedded within a project to which some authors were affiliated, we explicitly treated reflexivity as part of study rigor (Braun et al. 2022). The research team comprised four researchers, including three males and one female, with complementary expertise in health informatics, information systems, and qualitative health systems research. The diversity of the research team contributed to reflexive discussions throughout data collection and analysis and helped minimize individual interpretive bias. During data collection and analysis, the research team kept analytic notes on how project involvement could shape respondent expectations and researcher interpretation, and we discussed this as a limitation.

The Tanzanian health system is decentralized, and routine health data flow from facilities to the council and national levels through a mixture of paper tools and digital platforms (Kikoba et al. 2025). DHIS2 functions as the national aggregate routine data warehouse and analytics platform. GoT-HOMIS (Government of Tanzania Health Operation Management Information System) is a government electronic medical record and facility operations system used for patient-level management in many facilities (Mwogosi and Mambile 2025). In this article, DHIS2 and GoT-HOMIS are treated as linked but distinct systems: DHIS2 primarily supports aggregate reporting and analysis, whereas GoT-HOMIS primarily supports patient-level clinical and administrative workflows. We do not exclude platform-related constraints from the analysis; rather, we examine them as part of the technical and institutional conditions of data use.

### Sampling and participants

Twelve health facilities were purposively selected, with six facilities drawn from each study council. Facility selection was guided by three criteria: (i) active use of the District Health Information Software 2 (DHIS2) for routine health information reporting, (ii) variation in level of healthcare delivery, and (iii) geographical distribution across the two councils. The final sample comprised six dispensaries, four health centers, and two hospitals. Six facilities were located in Dodoma City Council, representing predominantly urban and peri-urban service settings, while the remaining six were drawn from Bahi District Council to capture a predominantly rural service context. Facilities were not selected based on performance rankings or reporting scores; rather, purposive sampling sought to achieve analytical variation across different organizational and service delivery contexts.

A total of 31 key informant interviews were conducted. Participants included facility in-charges, Health Management Information System (HMIS) focal persons, facility program coordinators, and council program coordinators as described in Table 1. Throughout this manuscript, the term “HMIS focal person role” was also referred to as the DHIS2 focal person or DHIS2 officer during fieldwork. These titles described substantially similar responsibilities, including coordinating routine health data collection, addressing data quality issues, overseeing DHIS2 data entry and verification, and generating routine reports. At the facility level, HMIS focal persons frequently held dual clinical and information management responsibilities, most commonly serving as nurses or clinicians in addition to their HMIS functions. At the council level, personnel with comparable designations coordinated data verification, quality assurance, and follow-up activities across health facilities (Table 1).

**Table 1:** KII participants by role.

Role	Dodoma city	Bahi district	Total
Facility in-charge	4	3	7
HMIS focal person	6	6	12
Facility program coordinators	3	2	5
Council program coordinators	4	3	7
Total	17	14	31

*Note.* The facility sample comprised 12 facilities: six dispensaries, four health centers, and two hospitals.

### Data collection

Data were collected through semi-structured key informant interviews and nonparticipant observations during repeated field visits in 2024 and early 2025. Interviews were conducted by the first and second authors, who also designed the interview guides. The guides focused on data flows, data use practices, roles and responsibilities, training, data quality checks, annual planning, targets, and experiences with digital platforms. All invited participants agreed to participate in the study, and no interviews were declined, withdrawn, or terminated before completion. Because data use practices were often embedded in routine work, observations were used to document how data moved through everyday settings. With participants’ informed consent, all interviews were audio-recorded to ensure accurate capture of participants’ accounts. Field notes were also taken during and immediately after interviews to document contextual observations, nonverbal cues, and emerging analytical reflections.

The observational component focused on data review meetings, printed dashboards and scorecards, notice-board displays, paper registers and tally sheets, manual aggregation practices, and the use of DHIS2 and GoT-HOMIS during routine reporting. Observational data were not treated as a separate dataset with stand-alone themes. Instead, they were used to clarify the origin of practices described in interviews and to corroborate whether such practices were materially visible in facilities and councils. For example, observations helped verify the use of printed performance displays, team-based data checks before submission, and the hand-adjustment of tally sheets when standard forms did not fit local reporting needs.

Potential bias was addressed in several ways. First, participants were informed that the study aimed to understand routine practice and that their responses would not affect project support. Second, all quotations used in reporting were anonymized by role and level of facility. Third, the team deliberately compared accounts across facilities and councils, including instances where respondents described disagreement, uneven uptake, or ongoing dissatisfaction. Quotations recorded in the Excel workbooks were lightly edited only for readability, where grammar obscured meaning; the substantive wording was preserved. Data collection continued until thematic saturation was achieved, that is, when no substantially new concepts or themes emerged from successive interviews. Consistent with the guidance of Guest et al. (2006), saturation was judged to have been reached when additional interviews yielded redundancy rather than novel insights, indicating that the major themes had been sufficiently developed (Guest et al. 2006). No repeat interviews were conducted, as each participant was interviewed once during the study period.

### Data analysis

Data were analyzed using a hybrid thematic approach. First, the first and second authors read interview notes, workbook excerpts, and observation notes repeatedly to familiarize themselves with the material. Second, an initial coding structure was developed deductively from PRISM, using organizational, behavioral/individual, and technical domains as broad containers (Aqil et al. 2009). Third, within these domains, the team coded inductively for recurring tensions in how participants described their work. At this stage, the analytical question shifted from asking whether a barrier existed to asking what conflicting expectations were being enacted and by whom, as presented in Table 2.

**Table 2:** Main contradictions and local institutional work.

Thematic area	Contradictory logics	Illustrative empirical finding	Dominant institutional work
Organizational	Accountability vs. resource control	Data review and follow-up were expected, but routine review budgets, printing, and action resources were unstable or held elsewhere.	Hybrid review meetings; reliance on existing management forums
	Expectation of data use vs. lack of standard operating procedures	Facilities were asked to use data in meetings and supervision, but there was no consistent template for indicators, formats, or assessments.	Locally improvised review routines and indicator selection
Individual	Primary clinical role vs. secondary HMIS responsibility	Nurses and other clinicians often doubled as HMIS focal persons.	Redistribution of tasks and partial shielding from other duties
	Distributed responsibility vs. concentrated capability	Many departments were expected to use data, but practical DHIS2 skills often rested with one trained person.	Peer training, champions, informal mentoring
Technical	System rollout vs. selective training	Platforms were in place, but training in analysis and interpretation was uneven.	Training-of-trainers and on-the-job coaching
	Standard tools vs. local data needs	Tally sheets and paper tools sometimes lacked required categories or space.	Hand-modified tools and local paper workarounds
	Data quality demands vs. weak digital DQA guidance	Facilities wanted consistent digital data but used *ad hoc* local checks.	Facility data teams and manual validation meetings
	Central targets vs. local denominator realities	Facilities adopted externally set targets that did not always fit catchment realities.	Pragmatic adoption with local interpretation
	Parallel platforms vs. integrated workflows	GoT-HOMIS, DHIS2, and paper registers required duplicate work and manual reconciliation.	Manual cross-checking, parallel use of systems, bypassing incomplete reports

Data coding and thematic analysis were conducted manually using Microsoft Excel and Microsoft Word to organize interview excerpts, observation notes, and emerging themes. Institutional theory informed interpretation in two linked ways. The first and second authors led the initial coding and theme development. The third and fourth authors reviewed the coding logic, challenged category boundaries, and discussed alternative interpretations. Disagreements were resolved through iterative discussion until consensus was reached on theme definitions and illustrative excerpts.

We identified contradictory institutional logics where participants described incompatible expectations, for example, being expected to use data while lacking procedural guidance or being held responsible for results without the resources to act on them. We then examined the responses to these contradictions through the lens of institutional work, distinguishing actions that created, maintained, or disrupted local arrangements around data use. The coding framework evolved iteratively from the initial PRISM domains to higher-order themes interpreted through institutional theory; a formal coding tree is not presented because coding categories were refined through repeated team discussions rather than a fixed hierarchical structure. Triangulation across interviews, observations, and the uploaded evaluation workbooks was used to improve interpretive rigor and to ensure that the final themes reflected both perceived and observed practice. Interview transcripts and preliminary findings were not returned to participants for member checking because the study focused on thematic interpretation across organizational settings rather than validation of individual accounts.

### Ethical considerations

This study was classified as institutional research focused on organizational systems, policies, and practices related to routine health data use rather than a human subject study on private clinical records or patient health status. Formal approval from a national health research ethics committee was therefore not required. Research clearance was obtained through the University of Dar es Salaam under the authority delegated to the Vice-Chancellor. Informed consent was obtained from all participants, and anonymity was maintained in reporting.

## Results

### Areas of data use

Across both councils, routine data were already being used for several management purposes: annual planning, budgeting, ordering medicines and supplies, outreach planning, community health education, and resource allocation. What varied was not whether data mattered but how data moved into action. Respondents described multiple local pathways through which data were made usable.

At facility level, a health center respondent in Dodoma explained that the quality improvement meeting had become a practical venue for reviewing departmental indicators: *“During QI meeting, we discuss indicators of the department … for example why we have poor performance in visit before 12 weeks … we discuss and find root causes”* (Health Center respondent, Dodoma). A dispensary respondent similarly described using routine data for community-facing work: *“data inform us about outbreaks of disease, and we communicate this during community meetings … we also use data for planning”* (dispensary respondent, Dodoma). At council level, one respondent in Bahi described how analysis of increasing malaria cases triggered follow-up to a swamp area and larvicide action, after which *“the number of cases was reduced”* (council respondent, Bahi).

These examples show that routine data use existed in practice. Yet respondents also made clear that such use was highly contingent on who had analytic skills, which meeting was currently available, which indicators could be generated from the systems, and whether facilities had resources to act.

### Contradictory logic around HIS and data use practices

Analysis of the interviews and observations revealed that routine data use was shaped by a series of contradictory institutional logics embedded within organizational arrangements, individual roles, and technical infrastructures. Rather than representing isolated barriers, these contradictions reflected competing expectations regarding service delivery, accountability, digitalization, and resource allocation that influenced how routine health information was generated, interpreted, and used in everyday practice. In response, actors developed various forms of institutional work to maintain routine data use under these conditions. Table 2 presents a summary of the identified contradictory logics, their empirical manifestations, and the corresponding institutional work observed across the study settings.

### Organizational contradictions

#### Accountability without commensurate resources

A recurring contradiction concerned the separation between responsibility and control over resources. Facility and council actors were expected to review data, respond to gaps, and demonstrate data use, but the resources needed to do so were unevenly distributed across levels. Participants referred not only to money but also to time, meeting space, supervision, printing of updated tools, internet bundles, and staff availability.

This contradiction was especially clear in discussions of review meetings and follow-up action. A council respondent in Dodoma noted: *“We don’t have stable and regular quarterly data review meetings due to financial resources; we sometimes add the agenda of data review in other meetings, such as facility in-charges meetings, and that makes it not enough dedicated time for discussing our data”* (Council respondent, Dodoma).

Here, accountability for discussing and acting on data remained in place, but the budget for routine review did not. At the facility level, respondents described using data to identify problems such as low outreach coverage or high anemia among pregnant women, yet action often depended on management approval or council-level support. Participants thus experienced accountability not as a simple requirement to submit reports but as a broader expectation to demonstrate data-informed action even when decision rights and operating resources were controlled elsewhere. This is why we interpret the issue as a contradiction rather than as a standalone resource shortage.

#### Data use expectations without standardized procedures

Facilities were expected to use data in community meetings, performance reviews, planning, and supportive supervision. However, respondents consistently described the absence of standard operating procedures or minimum data-use expectations that could tell them which indicators to present, in what format, at what frequency, and how such use should be assessed. In practice, local actors improvised. A dispensary respondent in Dodoma explained this clearly: *“We currently have immunization data when we attend these meetings; however, for other services, it just depends on the challenge at [a] particular moment, there are no formalities”* (Dispensary respondent, Dodoma). Similarly, the Dodoma council respondent *identified “lack of data use template or guidelines”* as a continuing challenge. The issue was therefore not that facilities were indifferent to data use; rather, they were expected to demonstrate it without a shared procedural script. This distinction matters analytically. The contradiction did not lie between data use and ignorance but between an institutional expectation that data should be routinely used and the absence of standardized routines for making such use legible and assessable across sites.

### Individual contradictions

#### Clinical roles versus secondary data responsibilities

A second contradiction concerned role design. In many facilities, the person responsible for routine data was also a clinician, nurse, or other service provider. Participants described data work as essential, yet it was commonly layered onto existing clinical responsibilities rather than allocated as a distinct position or protected function.

This arrangement produced a dual burden. Staff were expected to maintain patient flow, complete paper documentation, support digital entry, respond to council feedback, and generate analyses for meetings. The contradiction was not simply that staff were busy. It was that the system institutionalized data responsibility while continuing to organize staffing primarily around service delivery. One Bahi respondent therefore recommended that the HMIS focal role *“should be a position of its own and not … another assignment to a doctor or a nurse or pharmacist who also has other primary responsibilities”* (Council respondent, Bahi).

Participants also described the informal adjustments used to manage this tension. Some facilities tried to shield the HMIS focal person from selected extra duties, but this rarely removed them from clinical service altogether. Others distributed parts of the work informally across staff or relied on end-of-month team review sessions to catch up on aggregation.

#### Distributed responsibility versus concentrated capability

Reviewers correctly noted that the earlier manuscript treated the obligation to use data and the lack of DHIS2 knowledge as if they were automatically contradictory. Our revised interpretation is more precise: responsibility for data use was distributed widely across departments, but practical analytic capability was often concentrated in one or a few individuals. This mismatch created a contradiction between who was expected to use data and who could actually extract, validate, and interpret it. A Dodoma council respondent summarized this well by noting that one challenge was *“dependence on only one healthcare worker who has knowledge of entering data, so when they go on leave, challenges emerge”* (Council respondent, Dodoma). A facility respondent similarly argued that if more managers understood the system, they could *“do analysis by myself without depending on a person who is responsible for data”* (Dispensary respondent, Dodoma).

In other words, data use was framed institutionally as a shared managerial responsibility, but in practice, it depended on a narrow skills base. This capability concentration also shaped how contradictions were felt. When only one person could generate reports, others tended to experience DHIS2 as something adjacent to their work rather than integral to it. When more staff had been trained, respondents described stronger data ownership, easier error detection, and fewer calls from councils about discrepancies.

### Technical contradictions

#### System rollout versus selective training

Participants consistently described a gap between the rollout of systems and the breadth of user capability. DHIS2 was fully present in reporting routines, but training often reached only one focal person or a small subset of staff, with uneven follow-up on analysis and interpretation. A Bahi health center respondent said that the most beneficial capacity approach combined classroom exposure with facility mentorship because *“when we come back we have to train others”* (Health Center respondent, Bahi). Yet respondents also acknowledged the limits of such cascade arrangements. One facility suggested that training should involve *“more staff, at least 3 rather than only one designated focal person”* (Dispensary respondent, Dodoma).

These accounts indicate that the problem was not the absence of any training, but the institutionalization of a narrow training model in a setting where data use was expected to be broader than data entry. This helps explain why some facilities reported strong gains in ownership while still describing minimal routine analysis. Data entry can become decentralized more quickly than analytic competence.

#### Incomplete or outdated tools versus data needs

Participants also described mismatches between official tools and the data they were expected to capture. Changes in paper tools and digital templates were not always synchronized, and some tally sheets lacked enough space for categories encountered in practice. One dispensary respondent described the problem succinctly: *“Few spaces for tallying in ANC the age group of 14–20, which in reality has more clients”* (Dispensary respondent). Another noted that *“when MTUHA books changed, facilities had to print revised tools to capture data needed in DHIS2, which created an additional budget burden”* (Dispensary respondent, Dodoma).

These accounts were corroborated by field observations of hand-adjusted tally sheets and local modifications to paper forms. The contradiction here lay between standardized tools and variable service realities. Staff were expected to provide complete, category-specific data through tools that did not always fit the volume or structure of what they encountered clinically.

#### Data quality demands versus weak digital assessment guidance

Data quality was widely valued, but respondents described uncertainty about how digital data quality should be assessed across overlapping systems. At least some national guidance existed for data quality assessment, yet participants repeatedly stated that practical guidance for checking consistency across digital systems and for conducting routine local verification was weak or insufficiently operationalized.

A council respondent in Dodoma listed that DQA guidelines related to data collection and management in digital systems are missing among current challenges. At the facility level, actors responded by creating their own review arrangements. One Bahi health center respondent explained: *“We have a special data team representing each unit and an HMIS focal for reviewing data and submitting to DHIS. This team sits in a special meeting specific for data entry”* (Health Center respondent, Bahi). Another facility noted that data quality was assessed *“manually by sitting as a team”* (Dispensary respondent, Bahi).

These practices demonstrate that actors did not wait for ideal guidance. Instead, they created local verification routines. Yet the need to do so reveals a contradiction between the demand for increasingly consistent digital data and the lack of equally institutionalized guidance on how local teams should routinely verify those data.

#### Standardized targets versus local catchment realities

Coverage targets were often derived from council or national sources and then adopted locally. Respondents described this as necessary but problematic because denominators and annual expectations did not always reflect real catchment populations or service utilization patterns.

A dispensary respondent stated plainly: *“The targets are set at the council level; we do adopt them into our plans”* (Dispensary respondent, Dodoma). Another respondent explained that target quantification was *“very hard, especially setting the target and ceilings”* (Health Center respondent, Bahi). The practical effect was that facilities sometimes appeared to overperform or underperform for reasons tied less to service change than to denominator mismatch.

This was therefore not just a problem of technical calculation. It was a contradiction between centralized standardization and locally situated service realities. Indicators were meant to guide action, but their credibility weakened when staff perceived the denominators as externally imposed and insufficiently grounded in their catchment context.

#### Parallel systems versus integrated information needs

One of the clearest technical contradictions concerned the coexistence of DHIS2, GoT-HOMIS, and paper registers. Respondents frequently described duplicate recording, inconsistent data items, and missing report outputs. In principle, GoT-HOMIS and DHIS2 should be complementary: GoT-HOMIS captures patient-level operational data, while DHIS2 serves as the national aggregate warehouse. In practice, respondents experienced the relationship as fragmented because data still had to be re-entered or manually reconciled.

The phrase “double working” due to centralized GoT-HOMIS and DHIS2 appeared repeatedly across the evaluation workbook, including from hospital, health center, and dispensary respondents. One facility respondent noted that *“it takes more time to record each patient’s particulars in Centralized GoT-HOMIS and MTUHA, NHIF, etc.”* (Health center respondent, Dodoma). Another respondent reported that some data items were *“not found in GoT-HOMIS during generating report”* (Dispensary respondent). Observation notes and respondent accounts also indicated that certain planning tables still required manual aggregation because the expected DHIS2 report was incomplete or not aligned with current planning templates.

We therefore interpret DHIS2- and GoT-HOMIS-related challenges not as external to the study question but as central to it. The contradiction lies in expecting an integrated digital workflow while operationalizing data work through partially connected systems that still rely on paper reconciliation.

### Institutional work: how actors kept data use going

Local actors did not merely endure contradictions; they actively worked around them. Three patterns of institutional work were especially visible.

First, actors created new local arrangements. Review meetings were attached to existing performance forums when there was no dedicated budget for stand-alone data meetings. Peer training emerged where one trained focal person trained colleagues. Facilities formed *ad hoc* data teams to review consistency before submission, and within the DoE project, locally useful dashboards were developed around a set of 17 indicators that respondents found easier to retrieve and discuss than standard outputs. A Dodoma respondent described this shift practically: *“nowadays when we need data we extract/analyses from the system e.g. top ten diseases in different formats graphs, table, or pie charts”* (Dispensary respondent, Dodoma).

Second, actors maintained existing structures while adapting them. Hierarchical approval processes remained intact, and most facilities continued to rely on council-set targets. Existing staff were still appointed to data roles rather than replaced by dedicated data clerks. Paper registers, GoT-HOMIS, and DHIS2 continued to run in parallel because this was the only way to preserve reporting continuity. Such practices were not ideal, but they stabilized routine work.

Third, some responses had a disruptive character. When planning teams bypassed standard DHIS2 outputs and manually aggregated register data because key tables were missing, they implicitly challenged the adequacy of existing digital reports. Likewise, when locally designed dashboards became central to discussion, they disturbed the assumption that top–down, generic system outputs were sufficient for facility-level management. These actions did not overthrow existing institutions, but they exposed their limits and created pressure for revision.

## Discussion

### Synthesis of findings

This study shows that routine data use at the subnational level in Tanzania is neither absent nor fully institutionalized. Instead, it is improvised through local effort within an environment shaped by contradictory institutional logics. The main analytical value of this finding is that it clarifies why familiar RHIS barriers persist even where staff value data and use it in concrete ways. The issue is not only insufficient training, poor tools, or weak data quality in isolation. It is that local actors are expected to simultaneously satisfy competing logics of service delivery, reporting compliance, and digital standardization, with only partial alignment between them.

This interpretation extends existing work from Tanzania and elsewhere. Prior studies have documented limited analysis, weak supervision, inconsistent procedures, and resource shortages in Tanzania’s HMIS (Rumisha et al. 2020, Mboera et al. 2021, Kiwanuka et al. 2026). National work on DHIS2 has also shown that district managers appreciate the usefulness of the platform but continue to face constraints related to access, infrastructure, and analytic capability (Simba et al. 2022). More recently, research from Dodoma found that routine data use among primary healthcare managers was associated with organizational support, regular analysis, and the display of key indicators (Yusuph et al. 2024). Our study adds value by explaining how such organizational and technical conditions are lived as contradictions within everyday practice and how actors respond to them.

The findings also align with broader RHIS literature that emphasizes the interaction of technical, behavioral, and organizational determinants (Aqil et al. 2009, Hoxha et al. 2022). The systematic review by Hoxha et al. (2022) showed that RHIS data use in low- and middle-income countries is commonly constrained by shortages of resources, weak feedback, and limited capacity. Our results support those conclusions but also show that subnational actors do not simply fail under such conditions. They create hybrid arrangements that keep data moving into local action, even if those arrangements remain fragile. Similarly, Muhoza et al. (2022) found in Senegal that attitudes, social expectations, and perceived control shape RHIS data use. In our cases, perceived control was central: actors often believed data were important and usable, but the resources, routines, and cross-system alignment needed to act on them were not fully under their control.

A further contribution of this study is its explicit treatment of platform-related institutional challenges. DHIS2 and GoT-HOMIS themselves generate part of the difficulty but not in a purely technical sense. The problem is not only that multiple platforms exist. It is that digitalization has advanced faster than institutional alignment. GoT-HOMIS and DHIS2 occupy different positions in the information architecture, yet incomplete interoperability and misaligned reporting expectations shift reconciliation work onto frontline staff (Bossen et al. 2019). National assessments have similarly emphasized the ongoing challenge of harmonizing electronic medical records with DHIS2 and reducing siloed digital systems (Kikoba et al. 2019a, Kikoba et al. 2025). We therefore do not exclude platform-generated risk from the analysis; we locate it at the heart of the contradictions around data use.

The study also refines how data use should be understood empirically. A review by Byrne and Heywood (2023) reported the lack of actual examples of data use practices, but several respondents described strong examples of actual use: analyzing top 10 diseases, tracking maternal indicators, planning outreach, improving budget lines, or ordering medicines. Yet those same settings often lacked standardized procedures for selecting indicators, monitoring annual plans, or assessing whether data were being used well. This is why the manuscript distinguishes between the presence of data-informed action and the institutionalization of routine data use. Local action existed, but it often depended on champions, project support, or improvised meeting structures rather than on stable institutional routines.

For policy and practice, the findings suggest five priorities. First, councils and the Ministry should define minimum subnational procedures for data review, including core indicators, meeting cadence, and how routine data use should be documented and assessed (Somi et al. 2017). Second, data use capability should be broadened beyond a single focal person through facility-level multi-user training, simple analytic job aids, and protected learning arrangements. Third, digital health governance should prioritize practical interoperability between GoT-HOMIS and DHIS2 so that aggregate reporting does not depend on duplicate entry and manual cross-checking (Kikoba et al. 2019b). Fourth, facilities need locally credible targets and denominator guidance, especially for coverage indicators that repeatedly generate implausible outliers (Kiwanuka et al. 2026). Fifth, routine review meetings should be institutionalized within budgets rather than attached opportunistically to other forums.

Compared with studies that describe data use challenges in similar settings, the added value of this article lies in the combination of institutional theory, subnational qualitative evidence, and concrete respondent excerpts. Rather than presenting a list of barriers, it demonstrates how contradictory expectations are enacted by managers, coordinators, and frontline staff, and how those actors keep routine data use alive through institutional work. That perspective helps explain why some apparently “temporary” workarounds become normalized and why strengthening RHIS requires institutional as well as technical reform.

### Study contribution

The study makes three main contributions. First, it advances the conceptual understanding of routine data use by reframing commonly reported “barriers” as manifestations of contradictory institutional logics, thereby explaining why routine data use remains difficult despite widespread recognition of its importance and substantial investments in digital health information systems. Second, by drawing on institutional theory and the concept of institutional work, the study demonstrates that frontline health workers and managers are not passive recipients of institutional constraints. Instead, they actively create, maintain, and adapt local practices that enable routine data use under resource-constrained conditions. This perspective extends existing RHIS literature by highlighting the dynamic relationship between institutional structures and everyday data practices. Third, the study provides empirical evidence from decentralized health systems in Tanzania showing that the effectiveness of digital platforms such as DHIS2 depends not only on their technical functionality but also on the institutional arrangements that govern their use, including organizational routines, role design, governance mechanisms, and interoperability with complementary systems such as GoT-HOMIS.

### Limitations

This study has several limitations. First, the findings are grounded in two councils in Tanzania and are therefore not statistically generalizable; however, they offer analytically transferable insights for other decentralized health systems in low- and middle-income countries facing similar institutional and digital health governance challenges. Second, the study was conducted within the District of Excellence project, to which some authors were affiliated. That embedded position provided access and detailed knowledge of local processes, but it may also have shaped what participants emphasized, especially when discussing project-related tools such as dashboards and review meetings. We tried to reduce this risk through triangulation, anonymization, and explicit reflexive discussion within the team.

## Conclusion

This study demonstrates that routine health data use at the subnational level in Tanzania is not absent but is shaped by a complex institutional environment characterized by competing expectations surrounding service delivery, accountability, and digital reporting. Within these contradictory institutional logics, health workers and managers continue to use routine data for planning, budgeting, supportive supervision, resource allocation, disease surveillance, and service improvement. However, these practices are largely sustained through locally improvised arrangements rather than through standardized and institutionalized organizational processes. They did so by creating hybrid routines, maintaining workable but imperfect arrangements, and occasionally disrupting inadequate system outputs through local innovation.

These findings suggest that strengthening routine data use requires institutional as well as technological reform. Beyond expanding digital infrastructure, policymakers and health system managers should institutionalize standard procedures for data review and supportive supervision, strengthen analytical capacity across all facility staff rather than concentrating expertise in a single focal person, establish locally credible performance targets, clarify data-related roles and responsibilities, and improve interoperability between patient-level and aggregate reporting systems. Such reforms would reduce reliance on informal workarounds and support the sustained institutionalization of routine data use as an integral component of decentralized health system governance.

## Supplementary Material

Supplementary_materials_oqag036

## Data Availability

The data underlying this article are available in the article, the uploaded supplementary materials provided for revision, and associated online supplementary material, subject to confidentiality protections for participants.
